# Identification of a new allele of *BraA09g066480.3C* controlling the wax-less phenotype of Chinese cabbage

**DOI:** 10.1186/s12870-023-04424-3

**Published:** 2023-09-01

**Authors:** Chuanhong Liu, Longfei Yu, Lu Yang, Chong Tan, Fengyan Shi, Xueling Ye, Zhiyong Liu

**Affiliations:** 1 Laboratory of Vegetable Genetics Breeding and Biotechnology, Department of Horticulture, Shenyang Agricultural University, No. 120 Dongling Road, Shenhe District, Shenyang, 110866 China; 2 Vegetable Research Institute of Liaoning Academy of Agricultural Sciences, Shenyang, 110161 China

**Keywords:** Flowering Chinese cabbage, Epidermal wax, Bulked segregant analysis (BSA), Transcriptome sequencing

## Abstract

**Background:**

Epidermal wax covers the surfaces of terrestrial plants to resist biotic and abiotic stresses. Wax-less flowering Chinese cabbage *(Brassica campestris* L. ssp. *chinesis* var. *utilis* tsen et lee) has the charateristics of lustrous green leaves and flower stalks, which are of high commercial value.

**Results:**

To clarify the mechanism of the wax deficiency, the wax-less flowering Chinese cabbage doubled-haploid (DH) line ‘CX001’ and Chinese cabbage DH line ‘FT’, obtained from isolated microspore culture, were used in the experiments. Genetic analysis showed that the wax-less phenotype of ‘CX001’ was controlled by a recessive nuclear gene, named *wlm1* (*wax-less mutation 1*), which was fine-mapped on chromosome A09 by bulked segregant analysis sequencing (BSA-seq) of *B.rapa* genome V3.0. There was only one gene (*BraA09g066480.3C*) present in the mapping region. The homologous gene in *Arabidopsis thaliana* is *AT1G02205* (*CER1*) that encodes an aldehyde decarboxylase in the epidermal wax metabolism pathway. Semi-quantitative reverse transcription PCR and transcriptome analysis indicated that *BraA09g066480.3C* was expressed in ‘FT’ but not in ‘CX001’. *BraA09g066480.3C* was lost in the CXA genome to which ‘CX001’ belonged.

**Conclusion:**

The work presented herein demonstrated that *BraA09g066480.3C* was the causal gene for wax-less flowering Chinese cabbage ‘CX001’*.* This study will lay a foundation for further research on the molecular mechanism of epidermal wax synthesis in flowering Chinese cabbage.

**Supplementary Information:**

The online version contains supplementary material available at 10.1186/s12870-023-04424-3.

## Background

Epidermal wax is the grayish-white fatty substance that coats the surfaces of the above-ground parts of terrestrial plants [1]. Plant epidermal wax has a variety of ecological functions, such as preventing non-stomatal water loss from plants, maintaining plant surface cleanliness, resisting pests and diseases, and providing a protective barrier against biotic and abiotic stresses [2]. In addition, epidermal wax is also involved in various physiological functions such as fruit and leaf morphological development and preventing fruit cracking, and it has an effect on plant fertility [3]. As an important bolting vegetable, flowering Chinese cabbage is a famous specialty vegetable in China, and also has a large cultivated area and yield in South China. The stem is the main edible part of flowering Chinese cabbage. Stems lacking epidermal wax exhibit enhanced tenderness, rendering them highly favored by consumers and holding significant economic value.

The synthesis of cuticular wax begins in the plastids of epidermal cells [4]. Acylacetyl-CoA generates C16 and C18 fatty acids from the actions of fatty acid synthase and acetyl coenzyme A carboxylase. C16 and C18 fatty acids are catalyzed by long-chain acyl-CoA synthase (LACS) to generate acyl-CoAs, which are then transferred to the endoplasmic reticulum (ER) for extended synthesis to generate C20-C34 ultra-long-chain fatty acids, and then to synthesize very-long-chain fatty acids (VLCFAs). Derivatives of VLCFAs are generated in two ways, one of which is the acyl reduction pathway. VLCFAs can be reduced to primary alcohols by reductases, and the primary alcohols can further form cuticular waxes with fatty acids. The other way in which VLCFAs are derivatized is decarbonylation. Under the actions of reductases, fatty acids mainly produce alkanes, aldehydes, alcohols (primary and secondary), esters, fatty acids and ketones [5, 6]. After VLCFA derivatives were synthesized in the endoplasmic reticulum (ER), they were transported from the ER to the cell membrane, passed through the cell wall, and were secreted into the stratum corneum, forming cuticular wax [7].

Dellaert et al. [8] reported the first epidermal wax *eceriferum* (*cer*) mutant in *Arabidopsis thaliana*, and subsequently a great many genes involved in the regulation, biosynthesis, and transport of waxes were identified. Wax-related genes have been reported in many plant species such as maize (*Zea mays*) [9], barley (*Hordeum vulgare*) [10], cucumber (*Cucumis sativus*) [11], tomato (*Solanum lycopersicum*) [12], wheat (*Triticum aestivum*) [13], rice *(Oryza sativa*) [14], and sorghum (*Sorghum bicolor*) [15]. The epidermal wax-related gene *BnCER1* was mapped to A09 chromosome in *Brassica napus* [16] In cabbage, *BrWax1* was mapped to A01 chromosome [17]; *BoCER1* had a 2,722-bp insertion [2] and a 252-bp insertion [18] respectively, resulting in a wax-deficient phenotype. The *BrCER1* gene of the wax less mutant of non-heading Chinese cabbage had a 39-bp deletion [6]. In the *Brcer1* gene of Chinese cabbage, there was a single-base C to T transition which resulted in the loss of epidermal wax [19]. The gene responsible for the *wlm1* mutation has not been fine mapped in flowering Chinese cabbage.

In this study, scanning electron microscopy (SEM) observation showed that the surfaces of ‘CX001’ plants are smooth and are without wax. We mapped the wax-less gene *wlm1* in flowering Chinese Cabbage using BSA-Seq and found that the loss of *BraA09g066480.3C* on chromosome A09 resulted in plants with a wax-less, smooth-surface phenotype. Semi-quantitative RT-PCR analysis and transcriptome analysis showed that *BraA09g066480.3C* was not expressed in ‘CX001’. The genome sequence of CXA (the wax-less line) in the *Brassicaceae* Database indicated that the locus *BraA09g066480.3C* has been lost in flowering Chinese cabbage line ‘CX001’. This suggests that *BraA09g066480.3C* plays a key role in wax synthesis in flowering Chinese Cabbage.

## Materials and methods

### Plant materials

‘CX001’ is a doubled-haploid line of flowering Chinese cabbage, obtained from microspore culture isolated from ‘Super Grade 50 Sweet Flowering Chinese Cabbage’, with bright green leaves and stems. ‘FT’ is a DH line of Chinese cabbage, obtained from microspore culture isolated from ‘FT50’. The materials were planted in Shenyang Agricultural University. ‘CX001’ and ‘FT’ were used as parents to prepare F_1_, F_2_, and BC_1_ populations for use in genetic analysis and gene mapping. F_1_ crossed with ‘FT’ to get BC_1_P_1_, crossed with ‘CX001’ to get BC_1_P_2_. The plants were cultivated in a greenhouse at Shenyang Agriculture University. The seeds were germinated on infiltrated filter paper and then vernalized for 15 days in a 4-degree refrigerator. After vernalization, the seeds were planted in seedling trays and grown in the greenhouse. After 25 days, the seedlings were transplanted into nutrition bowls within the greenhouse. During the bolting stage, the presence of epicuticular wax on stems and leaves was visually assessed to determine its presence. Wax-covered plants exhibited a grayish-white appearance, while wax less plants had a glossy surface.

### SEM analysis

Buds, stems, leaves, and siliques of ‘CX001’ and ‘FT’ were harvested for SEM analysis. The leaves were cut into 0.5 cm squares and fixed overnight in pentanediol, dehydrated, frozen in liquid nitrogen, lyophilized, and plated with gold. The samples were observed with a Hitachi Regulus 8100 (Japan) scanning electron microscope.

### DNA and RNA isolation

Fresh leaves were harvested at the bolting stage for DNA and RNA extraction. The cetyltrimethylammonium bromide (CTAB) method [20] was used for DNA isolation. The concentration of DNA was adjusted to 50 ng/μL. RNA was extracted from ‘FT’ and ‘CX001’ using the RNAprep pure Plant Kit (Tiangen) following the manufacturer's instructions. Total RNA was reversed-transcribed into cDNA using the TransScript One-Step gDNA Removal and cDNA Synthesis Kit (Trans, Beijing, China).

### BSA by resequencing

For BSA-seq, we selected 50 wax-less F_2_ plants to construct the WL-pool. Genomic DNA was isolated from the parental lines and WL-pool, fragmented into 400 bp fragments by sonication. These fragments underwent end repair, adapter ligation, purification, and PCR amplification for library preparation. Following library construction, preliminary quantification was performed using Qubit 2.0, and insert fragment lengths were determined using Agilent BioAnalyzer 2100. The average fragment length was appropriate. qPCR accurately quantified the library’s effective DNA concentration to ensure quality. The library was then sequenced using the Novaseq 6000 platform (Illumina, San Diego, CA, USA), and the sequencing mode was PE150. The off-machine data was quality-controlled with FastQC v0.11.7 [21], and clean data was obtained after removing low-quality sequences and reads containing sequencing adapters. The clean reads were compared to *B*.*rapa* genome V3.0 Database (http://www.brassicadb.cn/#/Download/) using BWA v0.7.12 [22] and the duplicate results were removed by Picard v1.94, and SNP and InDel detection and annotation were performed based on the alignment results.

SNP and InDel detection was performed using GATK v3.8.1 UnifiedGenotyper [23]. The SNPs and InDels were further filtered using the following criteria: SNPs were filtered with ‘QD < 2.0 || FS > 60.0 || MQ < 40.0 || SOR > 3.0 || MQRankSum <  − 12.5 || ReadPosRankSum <  − 8.0 || QUAL < 30.0’, and InDels with ‘QD < 2.0 || FS > 200.0 || QUAL < 30.0 || ReadPosRankSum <  − 8.0’. Sites with missing data were excluded.

After SNPs and INDELs were called, the SNP-index was calculated to locate regions related to the wax-less trait. The SNP-index value was obtained by following the methods in ‘Genome sequencing reveals agronomically important loci in rice using MutMap’ [24].

### Fine mapping of *wlm1* gene based on InDel marker

Validation and further shortening of the candidate intervals of the BSA-seq-identified *wlm1* gene by InDel molecular marker linkage analysis. The sequences within the candidate intervals were downloaded from the *B.rapa* genome V3.0 database. InDel primer design was accomplished via utilization of Primer Premier 5.0 software [25]. Polymorphism screening between the two parents and the two mixed pools was performed using InDel primers. The screened polymorphic InDel markers were used to narrow down the candidate interval in the F_2_ population. Primer screening and population linkage analysis were performed using 10 μL system PCR reactions and agarose gel electrophoresis. The recombination values were converted to the distance of the genetic map (cM) according to Kosambi's mapping function [26].

### Transcriptome analysis

Stems of ‘FT’ and ‘CX001’ were selected for RNA extraction, with three replicates per sample. The integrity and purity of the total RNA were determined using the Bioanalyzer 2100 and the RNA 6000 nano LabChip kit. After passing quality inspection, a sequencing library was constructed that was sequenced on an Illumina Hiseq4000 instrument using the PE150 mode. The raw read data was transformed into nucleotide sequences by base calling. The data was preprocessed by removing reads with joints and those that contained poly-A and poly-G tracts, reads containing > 5% Ns, and low-quality reads in which > 20% of the bases had Q scores < 10. The clean data reads were aligned to the reference genome using Hisat2 software (http://ccb.jhu.edu/software/hisat2/index.shtml) to assemble the transcripts. Based on the Hisat2alignment results, the transcripts were assembled using StringTie v2.1.4 [27] and the expression levels of all genes in each sample were calculated. The criteria for identifying differentially expressed genes (DEGs) were q-value < 0.05 and |log2 fold-change|> 1. The gene expression levels were measured using FPKM through the Stringtie v2.1.4. Differential expression analysis was performed with DESeq2 [28].

A bidirectional clustering analysis of the union of DEGs and samples was performed using the Pheatmap v1.0.12 software package in R language, clustering based on the expression levels of the same gene in different samples and the expression patterns of different genes in the same sample, with Euclidean method used to calculate distances and complete linkage in hierarchical clustering performed.

For Gene Ontology (GO) enrichment analysis, we used topGO v2.40.0 [29] and found significant GO terms with a P-value < 0.05 using the hypergeometric distribution method.

For Kyoto Encyclopedia of Genes and Genomes (KEGG) pathway enrichment analysis, we used clusterprofiler [30] and found significant KEGG pathways with a *P*-value < 0.05 using the hypergeometric distribution method [31].

### Semi-quantitative reverse transcription PCR (RT-PCR)

RNA extracted from flowers, stems, leaves, siliques, buds, and roots of ‘FT’ and ‘CX001’ at the bolting stage were used for semi-quantitative RT-PCR analysis. The internal expression control was the ACT gene and primers for *wlm1-1* were designed to amplify cDNA fragment (Table S1). Each 10 μL PCR mixture contained 1 μL cDNA. There was an initial denaturation at 94 °C for 5 min, followed by 35 cycles of denaturation at 94 °C for 30 s, annealing at 54 °C for 30 s, and extension at 72 °C for 1 min. The amplified DNA fragments were examined by electrophoresis on a 2% agarose gel.

### Quantitative Real-Time PCR (qRT—PCR)

RNA extracted from stems of ‘FT’ and ‘CX001’ was reverse transcribed into cDNA. Quantitative real-time PCR (qRT-PCR) assays were performed on a QuantStudio 6 PCR system using Ultra SYBR Mixture dye (Kangwei Century, Beijing, China). The internal control for gene expression was the ACT gene. Three biological replicated and three technical replicated each. The 2^−ΔΔCt^ method was used to calculate relative expression levels.

## Results

### Morphological characterization and genetic analysis of wax-less flowering Chinese cabbage

**Fig. 1:**
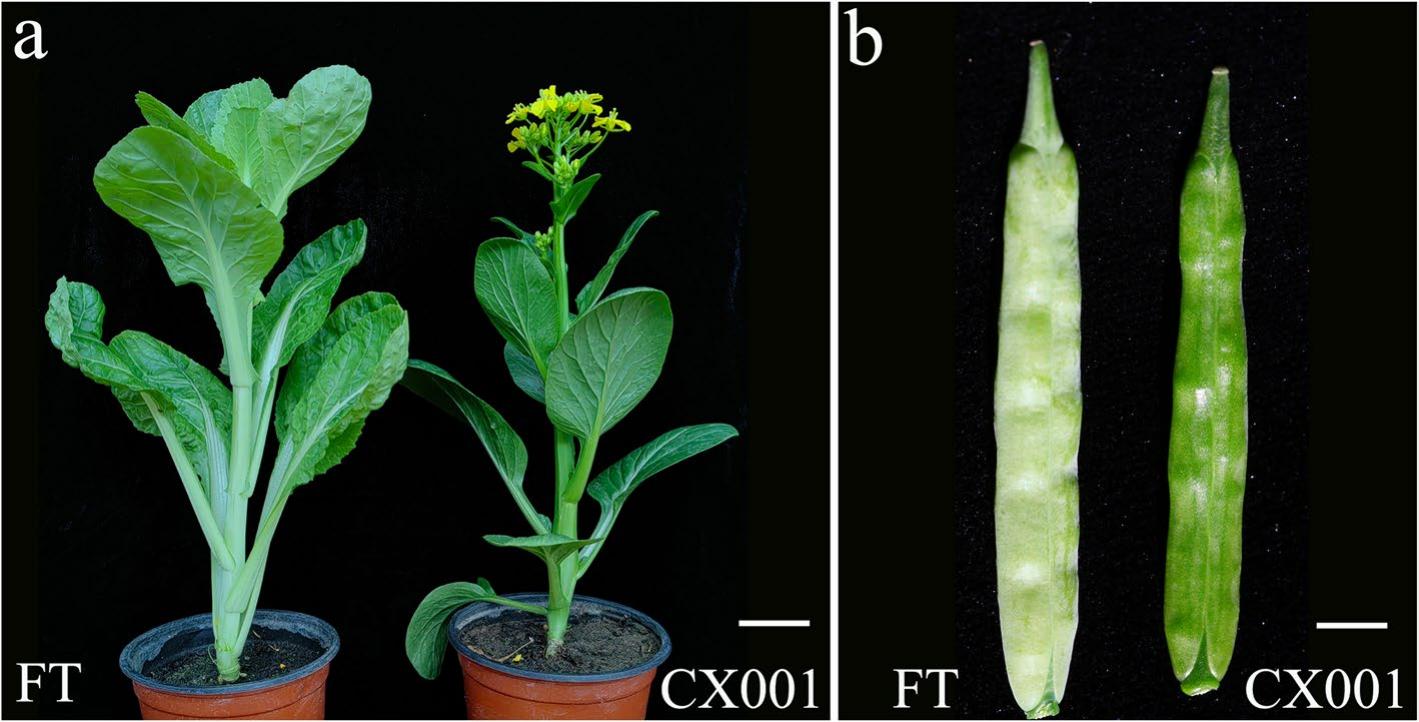
Plant phenotypes of ‘FT’ and ‘CX001’. **a** Plants of ‘FT’ and ‘CX001’ at the bolting stage. Bar = 4 cm. **b** Mature siliques of ‘FT’ and ‘CX001’. Bar = 6 mm

**Fig. 2:**
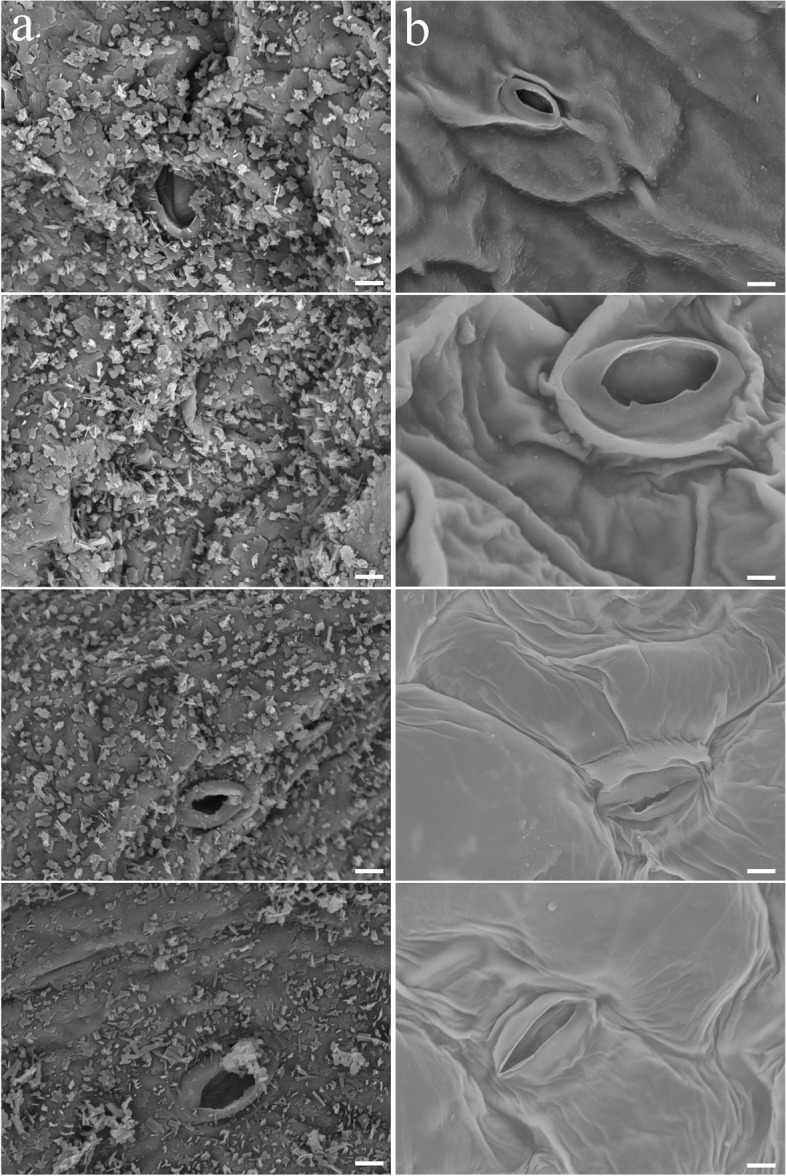
SEM observations were conducted on ‘FT’ and ‘CX001’. **a** Different parts of ‘FT’. From top to bottom were bud, stem, leaf and silique. **b** Different parts of ‘CX001’. From top to bottom were bud, stem, leaf and silique

Compared with the gray phenotype of ‘FT’ plants, the wax-less flowering Chinese cabbage plants exhibited a smooth green surface (Fig. 1a, b). SEM analysis was used to determine the structure of the wax on the surfaces of the buds, stems, leaves, and siliques of ‘FT’ and ‘CX001’. Our observations revealed that the epidermis of ‘FT’ plants showcased a substantial presence of columnar and flaky wax crystals (Fig. 2a), whereas ‘CX001’ plants exhibited an almost complete absence of wax crystals on their epidermal surfaces (Fig. 2b).

To investigate the heritability of the wax-less phenotype in ‘CX001’, the parental lines ‘CX001’ and ‘FT’ were crossed, and the F_1_ and F_1_′ generation plants all showed the gray epidermal phenotype, indicating that the wax-less trait from ‘CX001’ was controlled by one or more recessive nuclear genes. In a population consisting of 4,390 F_2_ plants, 3,287 were waxy and 1,103 plants showed the wax-less phenotype, with a waxy/wax-less segregation ratio of 2.98:1. A Chi-squared test showed that the segregation ratio in the F_2_ population fitted the expected segregation ratio of 3:1 (χ^2^ = 0.038 < χ^2^0.05 = 3.84) for a single recessive gene. The segregation ratio of the BC_1_P_2_ generation was 1:1 (χ^2^ = 0.186), and all of the BC_1_P_1_ progeny showed the waxy phenotype, indicating that the wax-less phenotype of ‘CX001’ was controlled by a recessive nuclear gene (Table 1).

**Table 1:** Genetic analysis in *wlm1*

Generation	Total	Waxy	Wax less	Segregation ratio	χ^2^	χ^2^_0.05_
P_1_ (FT)	30	30	0			
P_2_ (CX001)	30	0	30			
F_1_ (P_1_ × P_2_)	200	200	0			
F_1_′(P_2_ × P_1_)	200	200	0			
F_2_	4390	3287	1103	2.98:1	0.038	3.841
BC_1_P_1_ (F_1_ × FT)	163	163	0			
BC_1_P_2_ (F_1_ × CX001)	342	168	174	0.97:1	0.186	3.841

### Preliminary localization of *wlm1*

**Fig. 3:**
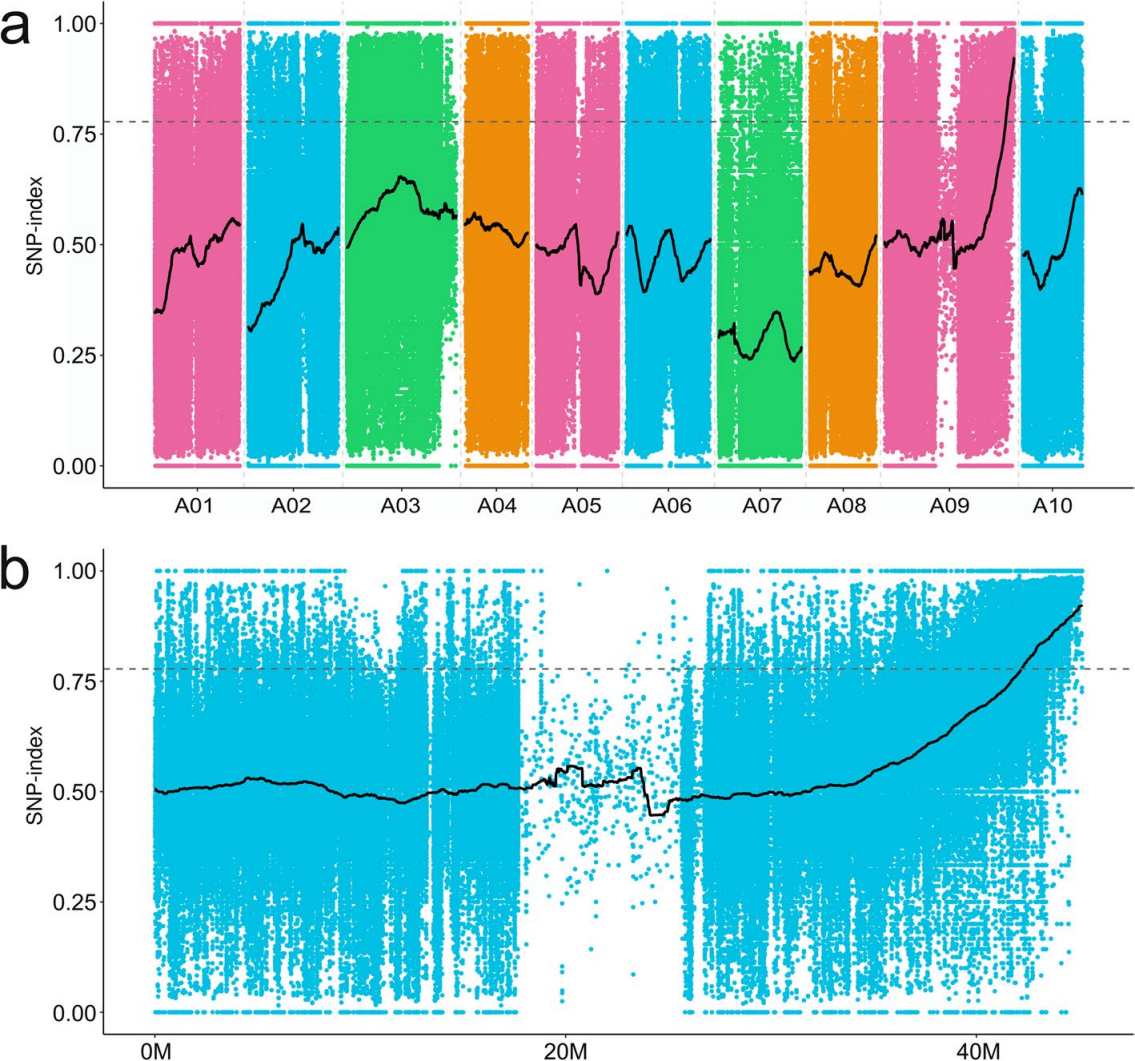
SNP-index positioning diagram. **a** Distribution of SNP-indexes on the 10 Chinese cabbage chromosomes. **b** The wax-less trait was mapped to a candidate gene localization region on chromosome A09. The dotted line represents the threshold value, which was selected based on the 99% quantile value of the black fitting curve

165529406, 144107158 and 160332,644 paired-end raw reads were obtained by high-throughput sequencer from WL-pool, ‘CX001’, and ‘FT’, respectively. After preprocessing raw reads, we generated 162,373,920, 136,686,604, and 157,381,608 valid reads (125-base) from the WL-pool, ‘CX001’, and ‘FT’, respectively. We used BWA [22] to align the reads to the reference genome, and the alignment results were de-duplicated with samtools v1.9. A total of 3,182,479, 2,378,095, and 1,812,142 reads remained after the WL-pool, ‘CX001’, and ‘FT’ were aligned to the reference genome. We analyzed the SNP and INDEL loci on the genome and obtained SNP and InDel information for each sample. A total of 3,323,198 SNP and 682,997 INDEL were obtained. To directly reflect the distribution of the SNP-index on the chromosomes of the mutant progeny, we made a location map (Fig. 3a). A sliding window calculation was performed based on the above results. With 90% as the standard, the candidate interval size was 29.49 Mb, distributed on chromosomes A03, A09 and A10 (Table S2, S3). With 95% as the standard, the candidate interval size was 14.78 Mb, distributed on chromosomes A03, A09 and A10 (Table S4, S5). The regions with SNP-index values in the top 1% were screened. From this analysis we identified a candidate genomic region for *wlm1* that extended from 42 to 45 Mb on chromosome A09 (Fig. 3b).

### Fine mapping of the *wlm1* gene

**Fig. 4:**
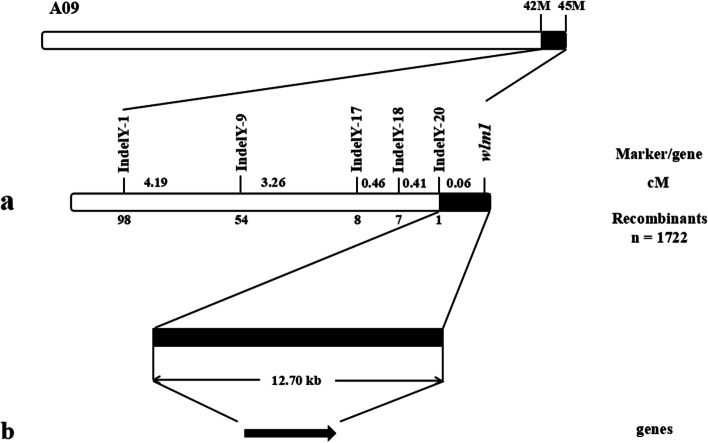
Genetic and physical maps of the *wlm1* locus in flowering Chinese Cabbage. (**a**) *wlm1* was mapped between the IndelY-20 locus and the end of chromosome A09. The 12.70 kb candidate region contained one gene. The numbers below the molecular marker loci represent the number of recombinants. The numbers to the right of the molecular marker loci represent the genetic distance between the marker and the candidate gene. (**b**) The 12.70 kb region containing *BraA09g066480.3C*. n: Population number of F_2_ generation wax-less plants

To further map the genetic position of the *wlm1* gene in flowering Chinese cabbage, 20 pairs of Indel marker primers (Table S6) were designed for the 3-Mb candidate genome region on chromosome A09. We screened 1,722 wax-less F_2_ plants and identified five polymorphic markers; IndelY-1, IndelY-9, IndelY-17, IndelY-18, and IndelY-20. The mapping results showed that the IndelY-1, IndelY-9, IndelY-17, IndelY-18, and IndelY-20 loci were on one side of *wlm1*, and the other side of *wlm1* was the end of chromosome A09. The *wlm1* gene was mapped to a region between the IndelY-20 locus and the end of the chromosomes. The genetic distance between the IndelY-20 and *wlm1* loci was 0.06 cM. The physical distance between the IndelY-20 locus and the end of chromosome A09 was 12.70 kb (Fig. 4). Only one gene, *BraA09g066480.3C*, was predicted to be present in the mapping region.

### Semi-quantitative RT-PCR analysis of *BraA09g066480.3C*

**Fig. 5:**
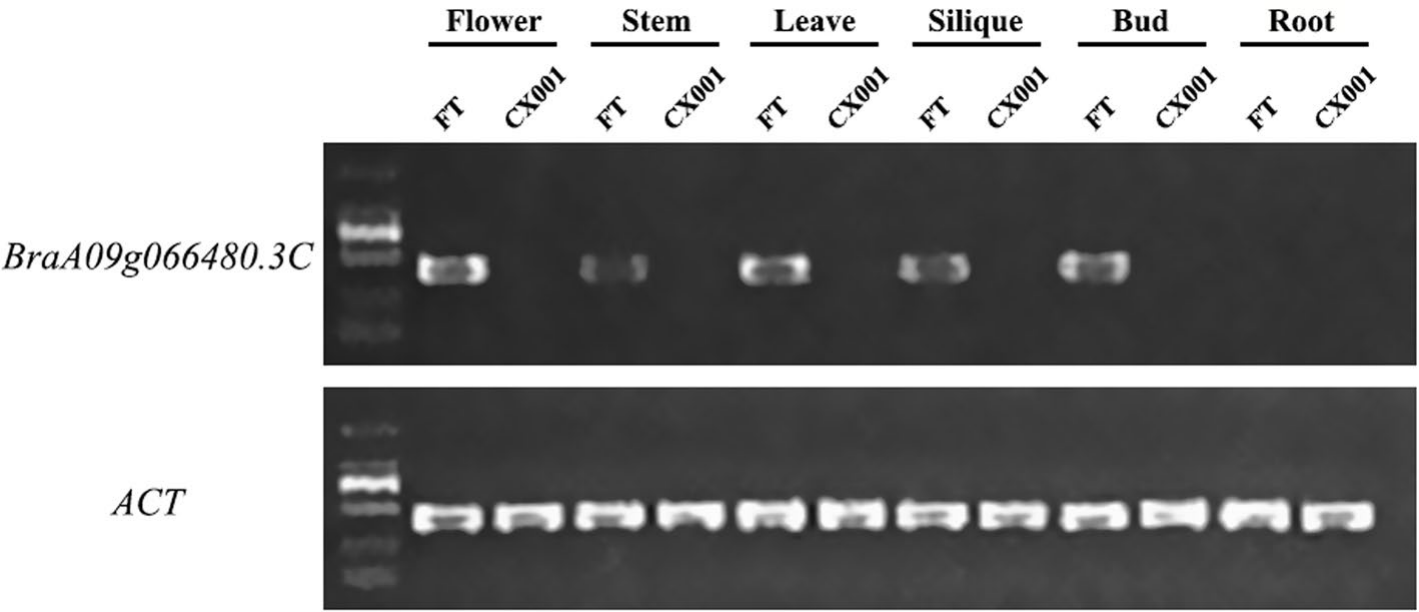
Semi-quantitative RT-PCR analysis of the expression of *BraA09g066480.3C* in flowers, stems, leaves, siliques, flower buds, and roots of ‘FT’ and ‘CX001’

cDNAs synthesized from RNA extracted from ‘CX001’ and ‘FT’ flowers, stems, leaves, siliques, buds, and roots were used as templates for semi-quantitative RT-PCR analysis. The *ACT* gene was used as the internal control for normalization of gene expression. The results showed that *BraA09g066480.3C* had the highest expression in flowers, followed by leaves, low expression in stem siliques and buds, no expression in roots of ‘FT’, while *BraA09g066480.3C* was not transcribed in the flowers, stems, leaves, siliques, buds, and roots of ‘CX001’ (Fig. 5, S1).

### Transcriptome analysis

Based on the transcriptome sequencing results, a total of 141,208,258 raw reads (21.32 Gb) were obtained from ‘CX001’, and 129,714,776 raw reads (19.59 Gb) were obtained from ‘FT’ (Table S7). The average ratios of Q30 for ‘CX001’ was 94.56%, and ‘FT’ was 94.37%. 120,734,332 and 131,186,308 clean reads remained for ‘CX001’ and ‘FT’, respectively. Among the six cDNA libraries, 91.82% (FT_1), 92.15% (FT_2), 92.56% (FT_3), 89.84% (CX001_1), 90.07% (CX001_2), and 90.04% (CX001_3) of the clean reads could be aligned to the reference genome, whose distribution localized to regions for CDS, Intron, Intergenic and UTR. The highest percentage of clean reads were aligned to CDS for ‘CX001’ and ‘FT’, with an average of 89.93% and 90.32%, respectively.

**Fig. 6:**
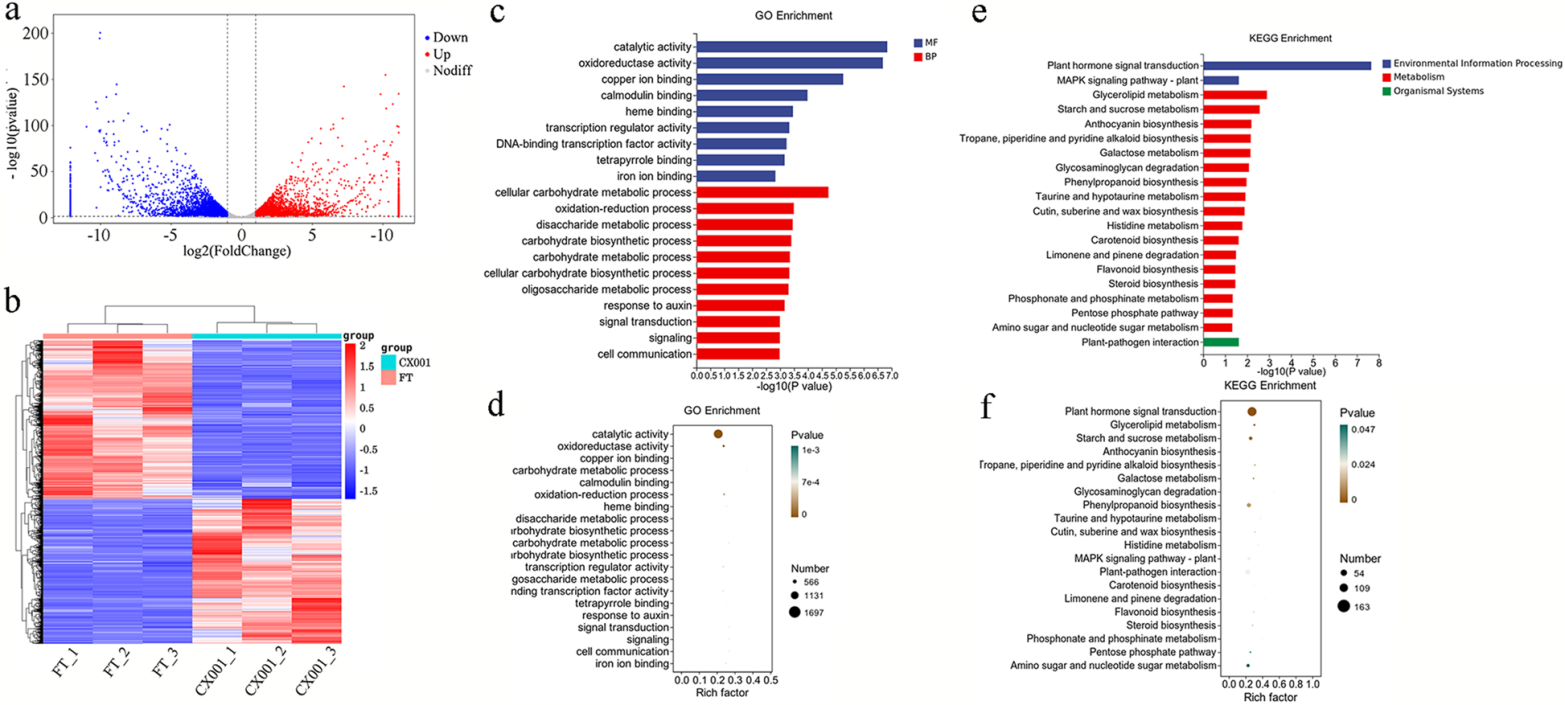
Transcriptome analysis of ‘FT’ and ‘CX001’. **a** Differentially expressed genes between ‘FT’ and ‘CX001’. **b** Cluster analysis of gene expression levels. **c** Histogram of GO enrichment results of DEGs between ‘FT’ and ‘CX001’. **d** Factor graph of GO enrichment results of DEGs between ‘FT’ and ‘CX001’. **e** Histogram of KEGG enrichment results of DEGs between ‘FT’ and ‘CX001’. **f** Factor graph of KEGG enrichment results of DEGs between ‘FT’ and ‘CX001’. We have obtained the appropriate copyright permission for KEGG images

A total of 8,202 DEGs were obtained from ‘FT’ and ‘CX001’, of which 3,927 genes were up-regulated and 4,275 genes were down-regulated in ‘CX001’ compared with ‘FT’(Fig. 6a). We conducted clustering analysis on DEGs, with red representing upregulated genes and blue representing downregulated genes (Fig. 6b). The darker the color, the higher the expression level. It is clear that the overall expression pattern of genes in the experimental group was significantly different from that of the control group, suggesting a significant inter-group difference and the grouping factor effectively separated the study individuals.

GO enrichment analysis was performed on 8,202 DEGs which were enriched in 1,700 GO terms (Table S8). Among the enriched GO terms, 1,024 were in the Biological Process domain, including ‘response to cellular carbohydrate metabolic process’ (GO: 0016491), ‘carbohydrate metabolic process’ (GO: 0005975), and ‘carbohydrate biosynthetic process’ (GO: 0016051), among others. There were 166 GO terms in the Cellular Component domain, which are mainly involved in ‘membrane’ (GO: 0016020), ‘integral component of membrane’ (GO: 0016021), and ‘photosynthetic membrane’ (GO: 0034357). There were 510 GO terms in the Molecular Function domain, including ‘catalytic activity’ (GO: 0003824), ‘oxidoreductase activity’ (GO: 0016491), and ‘heme binding’ (GO: 0020037) (Fig. 6c, d). *BraA09g066480.3C* was enriched in the ‘iron ion binding’ term (GO:0005506), ‘lipid biosynthetic process’ (GO:0008610), ‘oxidoreductase activity’ (GO:0016491) and ‘oxidation–reduction process’ (GO:0055114). Among these, the term ‘iron ion binding’ (GO:0005506) consisted of 98 DEGs, with 44 upregulated and 54 downregulated genes; the term ‘lipid biosynthetic process’ (GO:0008610) consisted of 40 DEGs, with 26 upregulated and 14 downregulated genes; the term ‘oxidoreductase activity’ (GO:0016491) consisted of 408 DEGs, with 201 upregulated and 207 downregulated genes; the term ‘oxidation–reduction process’ (GO:0055114) consisted of 344 DEGs, with 168 upregulated and 176 downregulated genes.

KEGG enrichment analysis showed that the DEGs were enriched to 127 pathways [32–34], and the main ones included ‘plant hormone signal transduction’ (brp04075), ‘cutin, suberine and wax biosynthesis’ (brp00073), ‘glycerolipid metabolism’ (brp00561), ‘starch and sucrose metabolism’ (brp00500), ‘anthocyanin biosynthesis’ (brp00942), ‘tropane, piperidine and pyridine alkaloid biosynthesis’ (brp00960), ‘galactose metabolism’ (brp00052) and ‘glycosaminoglycan degradation’ (brp00531) (Table S9) (Fig. 6e, f). *BraA09g066480.3C* was specifically enriched in the ‘cutin, suberine, and wax biosynthesis’pathway (brp00073), which contained a total of 19 differentially expressed genes. Among these genes, 7 were upregulated, namely *BraA09g024730.3C*, *BraA09g000630.3C*, *BraA04g026730.3C*, *BraA06g018310.3C*, *BraA07g028970.3C*, *BraA05g007850.3C*, and *BraA01g017490.3C*. Additionally, there were 12 downregulated genes in this pathway, specifically *BraA09g024730.3C*, *BraA09g000630.3C*, *BraA04g026730.3C*, *BraA06g018310.3C*, *BraA07g028970.3C*, *BraA05g007850.3C*, and *BraA01g017490.3C*.

**Fig. 7:**
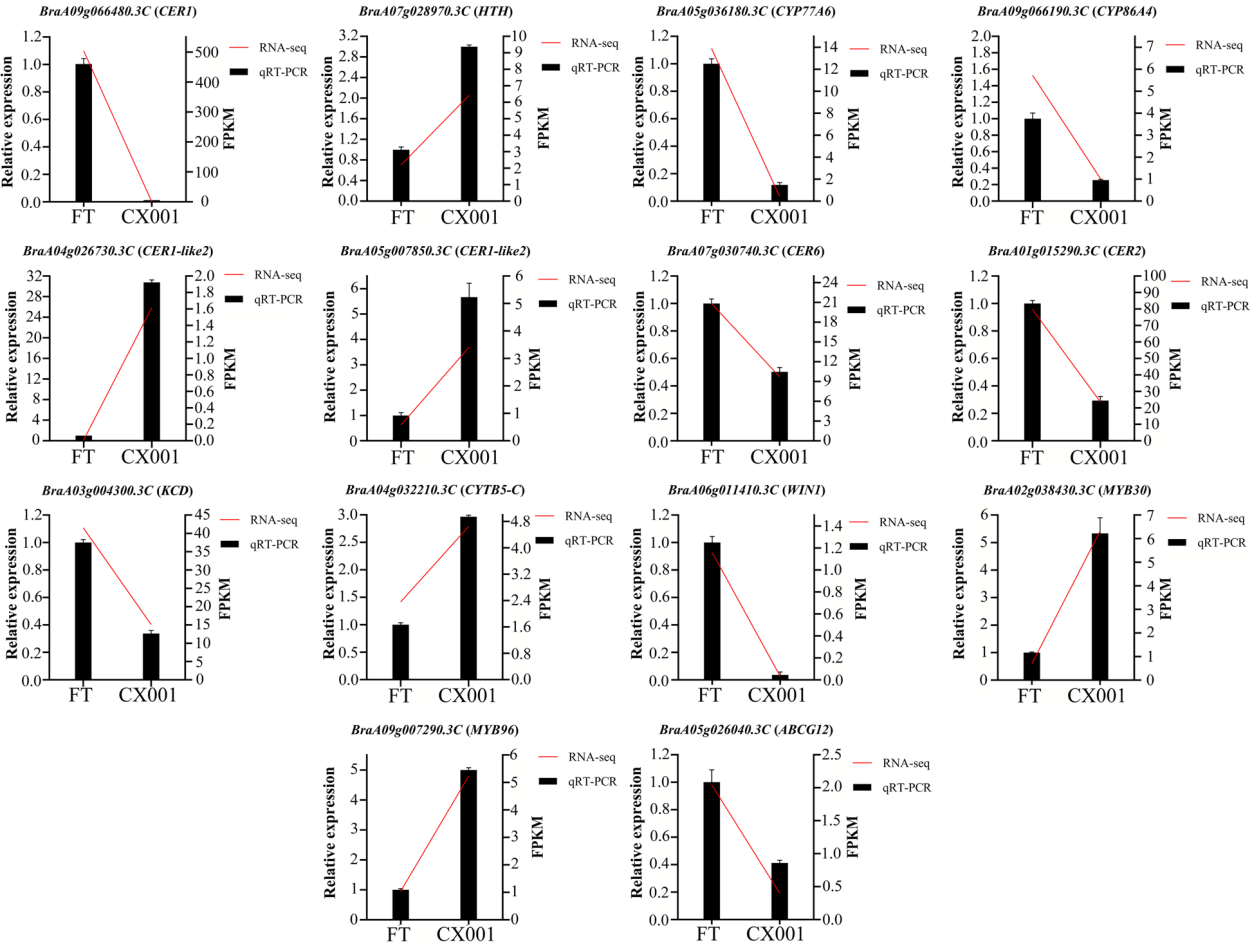
Expression pattern analysis of epidermal wax related DEGs between ‘CX001’ and ‘FT’

In the genes that were differentially expressed between ‘FT’ and ‘CX001’, genes involved in cuticular wax biosynthesis, cuticular wax transport, and transcriptional regulation were identified (Table S10). In the pathway of cuticular wax biosynthesis, the expression of candidate gene *BraA09g066480.3C* (*CER1*) in ‘FT’ was much higher than in ‘CX001’, with a maximum log2Fold-Change value of -10.2. Compared with ‘FT’, the expression of *BraA05g036180.3C* (*CYP77A6*), *BraA09g066190.3C* (*CYP86A4*), *BraA07g030740.3C* (*CER6*), and *BraA03g004300.3C* (*KCD*) was down-regulated in ‘CX001’; *BraA07g028970.3C* (*HTH*), *BraA04g026730.3C* (*CER1-like2*), *BraA05g007850.3C* (*CER1-like2*), *BraA01g015290.3C* (*CER2*), and *BraA04g032210.3C* (*CYTB5-C*) were up-regulated in ‘CX001’. *BraA06g011410.3C* (*WIN1*), *BraA02g038430.3C* (*MYB30*), and *BraA09g007290.3C* (*MYB96*) that belong to the transcriptional regulation pathway were differentially expressed in ‘FT’ and ‘CX001’. *BraA02g038430.3C* (*MYB30*) and *BraA09g007290.3C* (*MYB96*) were up-regulated and *BraA06g011410.3C* (*WIN1*) was down regulated in ‘CX001’. In the cuticular wax transport pathway, *BraA05g026040.3C* (*ABCG12*) was down regulated in ‘CX001’. qRT-PCR analysis showed that cuticular wax-related gene expression patterns were consistent with RNA-seq data, validating the accuracy and reliability of the transcriptome data (Fig. 7). The correlation coefficients between RNA-seq and qRT-PCR for the anterior to posterior genes in Fig. 7, were 0.99, 0.87, 0.98, 0.94, 0.94, 0.83, 0.78, 0.96, 0.96, 0.79, 0.97, 0.95, 0.90 and 0.88, respectively.

### Prediction and analysis of candidate genes

**Fig. 8:**
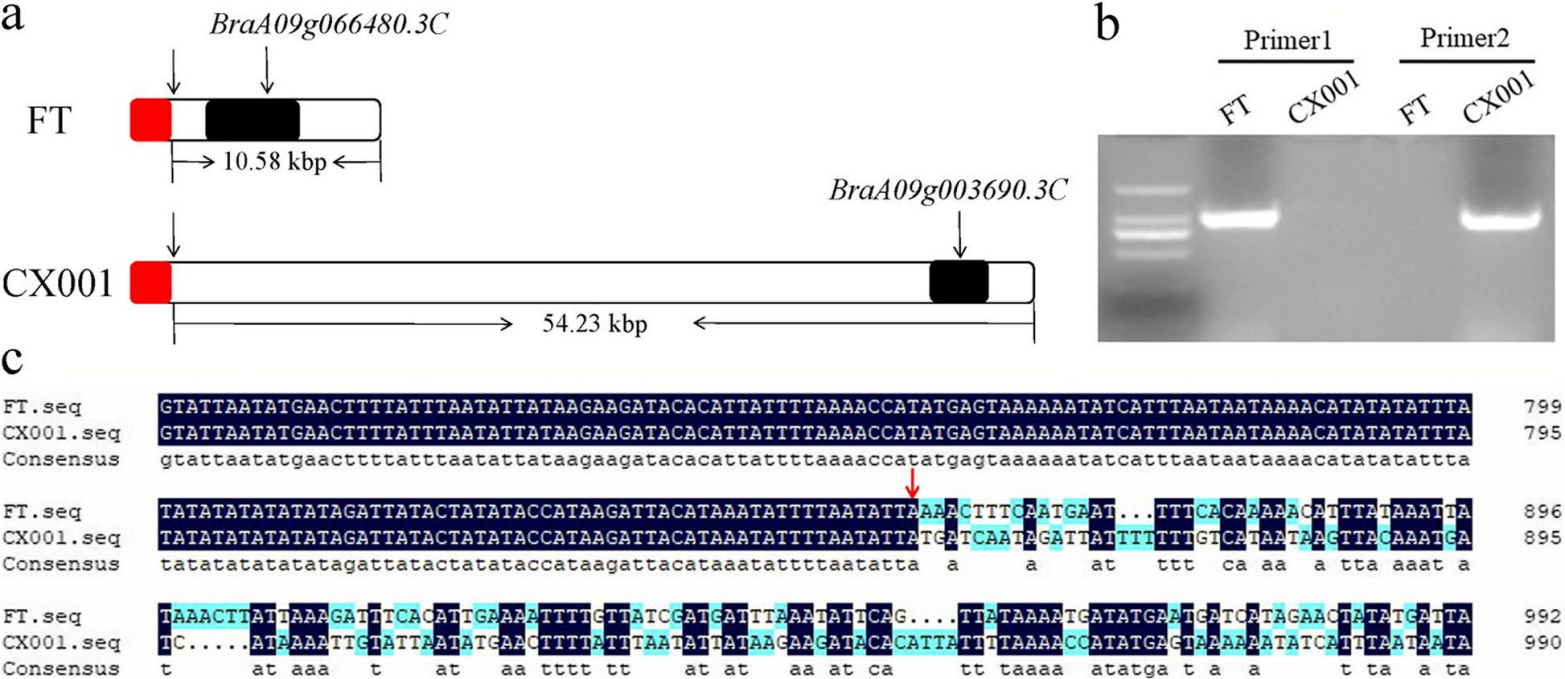
Differences between ‘FT’ and ‘CX001’ at the end of A09 chromosome. **a** Differences in *BraA09g066480.3C* at the end of chromosome A09 of ‘FT’ and ‘CX001’. The red part is the non mutated part. The right side of the red box is the mutation part. The white box is the intergenic region. Black boxes represent genes. The rightmost is the end of chromosome A09. **b** Agarose gel electrophoresis image of the primers amplification at both sides of the deletion sites in ‘FT’ and ‘CX001’. (**c**) Partial sequence information obtained from the amplification using Primer1 in ‘FT’ and Primer2 in ‘CX001’. The red arrow refers to position 45,146,224 on chromosome A09 in ‘FT’ and position 44,404,461 on A09 in ‘CX001’

**Fig. 9:**
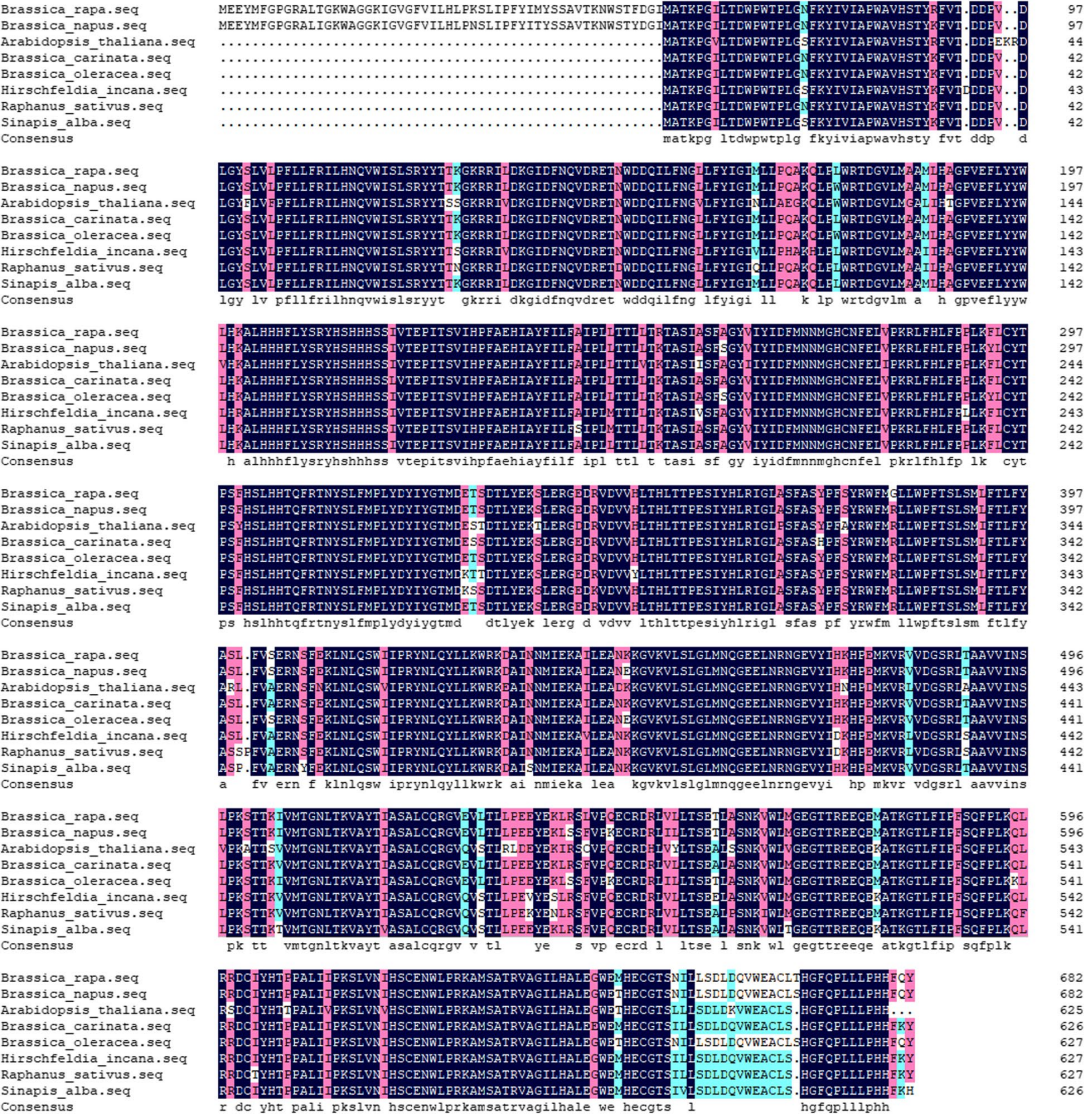
CER1 sequence alignment. Residues were color-coded to represent their degree of similarity. Black shading was used for residues with 100% similarity, pink shading for residues with ≥ 75% similarity, blue shading for residues with ≥ 50% similarity, and yellow shading for residues with ≥ 33% similarity

The *B*.*rapa* genome V3.0 Database (http://www.brassicadb.cn/#/Download/) and the *Arabidopsis* Information Resource (https://www.arabidopsis.org/) were used to analyze the *BraA09g066480.3C* gene within the mapping region, and the homologous gene in *Arabidopsis* is *AT1G02205* (*CER1*). In a study of *Arabidopsis cer1-1* mutant, it was found that the mutation of the *CER1* resulted in a smoother epidermis and greener color, and the composition of the waxy layer was also changed. The authors speculated that *CER1* may be related to enzymes involved in alkane synthesis [35, 36]. According to the CXA genome sequence completed by the Vegetable Institute of the Chinese Academy of Agricultural Sciences, a 10.58 kbp loss extending from position 44,404,461 to the end of the chromosome occurred on chromosome A09 of ‘CX001’. The deleted region was replaced with a sequence of 54.23 kbp in length; the lost sequence contains only one gene, *BraA09g066480.3C*, and the replaced sequence also contains only one gene, *BraA09g003690.3C* (Fig. 8a). Two pairs of primers, Primer1 and Primer2 (Table S1), were designed on both sides of the aforementioned deletion sites using the reference genomes of *Brassica rapa* and CXA, respectively. Our observations revealed that Primer1 amplified a band in ‘FT’, but not in ‘CX001’, while Primer2 did not generate a band in ‘FT’ but successfully amplified a band in ‘CX001’ (Fig. 8b). Sequencing of the amplified bands from ‘FT’ and ‘CX001’ revealed sequence variations occurring after position 45,146,224 on chromosome A09 in ‘FT’ and position 44,404,461 on chromosome A09 in ‘CX001’ (Fig. 8c), sequence information can be found in Table S11. *BraA09g066480.3C* gene amplification was successful in ‘FT’ but not in ‘CX001’, sequence information can be found in Table S11. Therefore, *BraA09g066480.3C* was predicted to be a possible candidate gene for *wlm1*. To investigate evolutionary conservation, we conducted a search for homologous proteins of CER1 in the National Center for Biotechnology Information (NCBI) database (https://www.ncbi.nlm.nih.gov) (Fig. 9). The highest homology of 97.51% was observed between CER1 in *Brassica rapa* and *Brassica napus.*

## Discussion

In this study, we determined that the wax-less trait observed in ‘CX001’ plants followed a typical Mendelian inheritance pattern, controlled by a pair of recessive nuclear genes. *wlm1* was mapped to within a physical region of 12.70 kb between position 45,144,111 and the end of chromosome A09, which included a single gene, *BraA09g066480.3C*. Functional annotation indicated that the homologous gene to *BraA09g066480.3C* in Arabidopsis was *CER1*, which encodes an aldehyde decarboxylase in the epidermal wax metabolism pathway. Transcriptome analysis and semi-quantitative RT-PCR showed that *BraA09g066480.3C* was not expressed in ‘CX001’. The above results were consistent with the sequencing results from the wax-less cabbage line ‘CXA’ in the *Brassicaceae* Database. The *BraA09g066480.3C* gene has been lost in the DH flowering Chinese cabbage line ‘CX001’. Therefore, we speculated that *BraA09g066480.3C* is the most likely candidate gene for *wlm1*.

Epidermal wax represents a crucial barrier between the aerial parts of the plant and the environment for terrestrial plants. The first report of wax-related mutants was in *Arabidopsis thaliana*; Dellaert et al. (1979) [8] found 53 epidermal wax mutants, of which the F4 mutant carried a mutation at the *cer-1* locus, and the wax-less trait was accompanied by semi-sterility. Koornneef et al. (1989) [37] elucidated the influence of environmental humidity on the fertility of epidermal wax mutants, namely *cer1*, *cer3*, *cer6, cer8*, and *cer10*. These mutants exhibited partial sterility under conditions of low humidity and near-complete sterility under conditions of high humidity. McNevin et al. (1993) [38] used T-DNA insertion to identify 13 *Arabidopsis thaliana eceriferum* mutants, including *cer1*. Wang et al. (2017) [6] cloned the mutant gene *Brcer1* from the epidermal wax mutant of non-heading Chinese cabbage. A mutation in *Brcer1* was also reported in Chinese cabbage, and it resulted in the loss of epidermal wax [19]. In our study, plants of the wax-less flowering Chinese cabbage line ‘CX001’ had glossy surfaces and lacked epidermal wax.

Aarts et al. (1995) [35] reported that the CER1 protein is an aldehyde decarboxylase which catalyzes the conversion of aldehydes to alkanes during epidermal wax synthesis. The CER1 protein has two conserved domains, stearoyl-CoA desaturase and fatty acid hydroxylase. The co-expression of *CER1*, *CER3*, and *MAH1* in siliques, flowers, stems, leaves, and seedlings suggests that they play a crucial role in the epidermal wax formation pathway [37]. In the *cer1* mutant plants, a significant reduction in the components of epidermal wax was observed, while a slight increase in the content of aldehydes was noted [18, 39, 40]. There are two ways to synthesize derivatives of VLCFAs in wax powder synthesis: decarbonylation and acyl reduction. The *cer1* mutation in flowering Chinese cabbage may lead to the failure of aldehydes to be converted to alkanes in the decarbonylation pathway, which further leads to the failure of alkanes to participate in the synthesis of secondary alcohols and ketones. SEM observation showed that there was a thick layer of epidermal wax on the surface of ‘FT’ plants, while the buds, stems, leaves, and siliques of ‘CX001’ were very smooth, with almost no wax powder, and the stomata were clearly visible.

*CER1* genes have been reported in many species. Hu et al. (2009) [41] cloned the CDS of *TaCer1* from wheat for the first time. Alkio et al. (2012) [42] showed that *PaCER1* was involved in the formation of epidermal wax in sweet cherry. *CER1-1* played a key role in citrus fruit wax formation [43]. *CsCER1* was involved in VLCFA biosynthesis in cucumber [44]. Qi et al. (2018) [45] cloned *MdCER1* from apple and found that it played an important role in the epidermal wax synthesis pathway. Similarly, *CER1* alleles have also been studied in species of *Brassica*. In cabbage, *Bol018504* had a 2,722-bp insertion [2] and a 252-bp insertion [18] respectively, resulting in a wax-deficient phenotype. *Bol018504* is homologous to CER1. The *Brcer1* gene of the wax less mutant of non-heading Chinese cabbage had a 39-bp deletion [6]. In the *Brcer1* gene of Chinese cabbage, there was a single-base C to T transition, the mutation site is located on the fourth exon, causing the conformation of the 255th amino acid to change from hydroxyl to benzene ring, and the amino acid sequence to change from S to F, that is, from serine to phenylalanine, which resulted in the loss of epidermal wax [19]. We found that *wlm1* was lost in the flowering Chinese cabbage line ‘CX001’. During species evolution, a loss of the *wlm1* gene occurred in ‘CX001’, but it did not have any discernible impact on the growth and development of the plants. Furthermore, this genetic alteration resulted in the desirable trait of wax-less in the individuals. and this is the first report of gene loss in flowering Chinese cabbage.

In summary, *wlm1* was fine mapped to a 12.70 kb region of chromosome A09, and the only gene in this region, *BraA09g066480.3C*, was lost from the genome of the flowering Chinese cabbage line ‘CX001’. *BraA09g066480.3C* was predicted to be the candidate gene for *wlm1*. *BraA09g066480.3C* encodes an aldehyde decarboxylase, which is a key enzyme in the epidermal wax biosynthesis pathway. The results of this study will extablishy a foundation for further research on the molecular mechanism of epidermal wax synthesis in flowering Chinese cabbage and other species in the *Brassicaceae*.

## Conclusions

We mapped the wax-less gene wlm1 in flowering Chinese cabbage and found that the loss of *BraA09g066480.3C* on chromosome A09 resulted in plants with a wax-less, smooth-surface phenotype. Transcriptome analysis and semi-quantitative RT-PCR showed that *BraA09g066480.3C* was not expressed in wax-less ‘CX001’. *BraA09g066480.3C* was lost from the genome of the flowering Chinese cabbage line ‘CX001’. This study will lay a foundation for further research on the molecular mechanism of epidermal wax synthesis in flowering Chinese cabbage.

### Supplementary Information

**Additional file 1:** **Table S1.** PCR primer sequences used for semi-quantitative RT-PCR, qRT-PCR, Primer1,Primer2 and the amplification of BraA09g066480.3C. **Table S2.** Region of 90% confidence interval. **Table S3.** SNPof 90% confidence interval. **Table S4.** Region of 95% confidence interval. **Table S5.** SNPof 95% confidence interval. **Table S6.** PCR primers used to amplify InDel markers in the candidate region. **Table S7.** Statistical trancriptome data. **Table S8.** DEGs in diferent enriched GO terms. **Table S9.** KEGG enrichment analysis of the DEGs. **Table S10.** Epidermal wax-related differentially expressed genes from ‘FT’ and ‘CX001’. **Table S11.** The sequence information obtained from amplification in‘FT’ and ‘CX001’.

**Additional file 2:** **Fig. S1.** Original picture of Fig. 5.

**Additional file 3:** **Fig. S2.** Original picture of Fig. 7b.

## Data Availability

The datasets supporting the conclusions of this article are included within the article and its additional files. The Illumina RNA-Seq datasets are available in the Gene Expression Omnibus (GEO) of the National Center for Biotechnology Information (NCBI) under the accession number GSE221778. The BSA-seq datasets are available in the the Sequence Read Archives (SRA) of the NCBI under BioProject ID: PRJNA916276. Genomic sequences and gene annotation information of *B*.*rapa* are downloaded online at http://brassicadb.cn.
