# Supplementary figures and images for: Identification of a new allele of BraA09g066480.3C controlling the wax-less phenotype of Chinese cabbage
Source: BMC Plant Biol. 2023 Sep 1;23:408. doi: 10.1186/s12870-023-04424-3 (PMC10472645; doi:10.1186/s12870-023-04424-3)

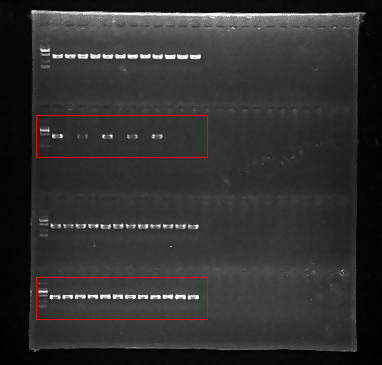

Supplement: Supplementary file 2 — Additional file 2: Fig. S1. Original picture of Fig. 5. [file 12870_2023_4424_MOESM2_ESM.tif]

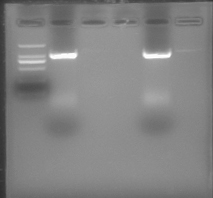

Supplement: Supplementary file 3 — Additional file 3: Fig. S2. Original picture of Fig. 7b. [file 12870_2023_4424_MOESM3_ESM.jpg]
